# A New Mechanistic Scenario for the Origin and Evolution of Vertebrate Cartilage

**DOI:** 10.1371/journal.pone.0022474

**Published:** 2011-07-22

**Authors:** Maria Cattell, Su Lai, Robert Cerny, Daniel Meulemans Medeiros

**Affiliations:** 1 Department of Ecology and Evolutionary Biology, University of Colorado, Boulder, Colorado, United States of America; 2 Department of Zoology, Charles University in Prague, Prague, Czech Republic; Ecole Normale Supérieure de Lyon, France

## Abstract

The appearance of cellular cartilage was a defining event in vertebrate evolution because it made possible the physical expansion of the vertebrate “new head”. Despite its central role in vertebrate evolution, the origin of cellular cartilage has been difficult to understand. This is largely due to a lack of informative evolutionary intermediates linking vertebrate cellular cartilage to the acellular cartilage of invertebrate chordates. The basal jawless vertebrate, lamprey, has long been considered key to understanding the evolution of vertebrate cartilage. However, histological analyses of the lamprey head skeleton suggest it is composed of modern cellular cartilage and a putatively unrelated connective tissue called mucocartilage, with no obvious transitional tissue. Here we take a molecular approach to better understand the evolutionary relationships between lamprey cellular cartilage, gnathostome cellular cartilage, and lamprey mucocartilage. We find that despite overt histological similarity, lamprey and gnathostome cellular cartilage utilize divergent gene regulatory networks (GRNs). While the gnathostome cellular cartilage GRN broadly incorporates *Runx*, *Barx*, and *Alx* transcription factors, lamprey cellular cartilage does not express *Runx* or *Barx*, and only deploys *Alx* genes in certain regions. Furthermore, we find that lamprey mucocartilage, despite its distinctive mesenchymal morphology, deploys every component of the gnathostome cartilage GRN, albeit in different domains. Based on these findings, and previous work, we propose a stepwise model for the evolution of vertebrate cellular cartilage in which the appearance of a generic neural crest-derived skeletal tissue was followed by a phase of skeletal tissue diversification in early agnathans. In the gnathostome lineage, a single type of rigid cellular cartilage became dominant, replacing other skeletal tissues and evolving via gene cooption to become the definitive cellular cartilage of modern jawed vertebrates.

## Introduction

A defining feature of the craniate subphylum (vertebrates and hagfish) is the “New Head”, which has been linked to the evolution of active predation in the vertebrate lineage[1]. The vertebrate head is a composite structure consisting of paired sense organs, an expanded central nervous system, peripheral ganglia, and a muscular, pumping pharynx. These components are physically supported and protected by cellular cartilage, which is replaced by bone in osteichthian vertebrates.

While it can be considered a vertebrate-specific morphological unit, most components of the vertebrate head have clear invertebrate antecedents. For instance, the basic organization of the vertebrate brain has deep roots in the neural tube of invertebrate chordates [2], [3], while the paired sense organs and cranial ganglia appear to be derived from evolutionarily ancient cranial placodes and sensory cells[4], [5], [6]. Similarly, the vertebrate pharynx, though modified for pumping water over the gills and capturing prey, retains the basic respiratory and feeding functions of the pharynx in all deuterostomes.

Although much of the vertebrate head likely evolved via the reorganization and augmentation of simpler precursors, the origin of cellular cartilage is less clear. Invertebrates appear to lack any tissue displaying the combination of morphological and biochemical properties that defines vertebrate cellular cartilage. In addition, comprehensive analysis of gene expression in amphioxus, a basal chordate, suggests that no single invertebrate cell type coexpresses all, or most, of the genes needed to drive cellular cartilage formation[7]. Rather, individual components of the vertebrate cartilage gene regulatory network (GRN) are expressed in different tissues, most of which are mesoderm-derived.

On the other side of the invertebrate/vertebrate transition, all extant vertebrates appear to possess bona fide cellular cartilage, with no tissue constituting an obvious evolutionary intermediate. Consistent with this, work from mouse, zebrafish, and frog suggests that vertebrate cellular cartilage development is mediated by a tightly conserved GRN[8], [9], [10], [11], [12], [13], [14], [15], [16]. In the head, the vertebrate cartilage GRN is initiated in migrating cranial neural crest cells (CNCC) by the transcription factors *SoxE*, *SoxD, Twist, tfap2, Ets*, and *Id*. After migration, CNCCs activate markers of nascent chondrocytes, including *Barx* [9], [17], *Runx* [10], [18], [19], [20], [21], and *Alx*/*Cart1* [22], [23], [24], [25]transcription factors. *Barx* and *Runx* then work with *SoxE* and *SoxD* to drive cartilage differentiation, partly by activating expression of the structural proteins Col2a1 (fibrillar collagen)[26], [27], [28] and Aggrecan[29], one of several chondrotin sulfate proteoglycans (CSPGs) expressed in vertebrate cartilage. In the trunk, a similar GRN is activated in mesodermal cells, though its initiation involves the transcription factors *Bapx* and *Pax1/9* [30], [31], [32]. Conservation of the cartilage GRN in neural crest and trunk mesoderm, and the presence of pharyngeal skeletons in invertebrate deuterostomes and fossil chordates, suggest that cellular cartilage first arose in the pharynx and later expanded into the head and trunk [1], [33], [34], [35], [36].

Classical and modern studies suggest that the core features of cellular cartilage development are conserved in the most basal extant vertebrates, the jawless agnathans. The branchial basket cartilage of the agnathan lamprey possesses all of the diagnostic histological and biochemical properties of gnathostome cellular cartilage including stack-of-coins and polygonal morphology, alcian-blue reactivity and fibrillar collagen expression [37], [38], [39], [40], [41], [42], [43], [44]. Modern ablation and vital dye labeling show that both gnathostome and lamprey pharyngeal cartilage is derived from cranial neural crest cells (CNCCs)[45], [46]. Furthermore, lamprey CNCCs coexpress many components of the gnathstome cartilage GRN, including *tfap2, Id, Twist, Ets, SoxD*, and *SoxE* [39], [47], [48], [49], [50].

While lamprey branchial basket cartilage is likely homologous to definitive gnathostome cellular cartilage, different regions have different properties. The “hard cartilage”[42], [43], [51] in the dorsal portion of the branchial basket skeleton consists of disorganized polygonal chondrocytes and expresses an elastin-like molecule called Lamprin [37], [52], [53]. In the branchial and hypobranchial bars, discoidal chondrocytes expressing fibrillar collagen and elastin generate so-called “soft cartilage”[38], [42], [43], [54]. Lamprey also possesses skeletal tissues with no clear relationship to gnathostome cartilage. Lamprey “mucocartilage” is the main skeletal tissue in the ventral pharynx and oral region[42], [43], [55]. While biochemically similar to definitive cellular cartilage [42], [51], mucocartilage is histologically distinct, consisting of scattered mesenchymal cells embedded in a mucopolysaccharide matrix[42], [55].

To better understand the evolutionary relationships between agnathan skeletal tissues and gnathostome cartilage, we are analyzing the expression of gnathostome cartilage GRN components in the sea lamprey *Petromyzon marinus*. Here we describe the expression of lamprey homologs of three key regulators of gnathostome chondrogenesis, *Runx*, *Barx* and *Alx/Cart1* from embryonic through larval stages when differentiated cellular cartilage is histologically discernable. Contrary to our expectations, we find that neither *Runx* nor *Barx* are co-expressed with *SoxE* genes in pharyngeal CNCC destined to form the cellular cartilage of the branchial basket. Rather, we see *Runx* expression in mesenchyme flanking the mouth, and *Barx* expression in the lower lip and mesenchyme medial to the pharyngeal mesoderm. Similarly, we see restricted expression of *Alx* in the dorsal and ventral aspects of the branchial basket and in the upper lip, but not in other *SoxE* and fibrillar collagen-expressing skeletal tissues. Given that *Runx*, *Barx*, and *Alx* are all required for gnathostome cellular cartilage development, our data show that lamprey and gnathostome cellular cartilage develop using divergent GRNs. Furthermore, combinatorial expression of lamprey *SoxE*, *Runx*, *Alx*, and *Barx* in different populations of presumptive mucocartilage suggest lamprey possess multiple skeletal tissues with genetic features of gnathostome cellular cartilage. Based on these findings, and previous work, we propose a scenario in which early agnathan vertebrates possessed an array of genetically and functionally distinct CNCC-derived skeletal tissues. In the gnathostome lineage, only one of these ancestral tissues was retained and evolved via gene cooption to become the cellular cartilage of modern gnathostomes.

## Results and Discussion

### *SoxE* expression, type A fibrillar collagen expression, and alcian blue staining support common evolutionary and developmental origins for mucocartilage and definitive cellular cartilage

**Figure 1:**
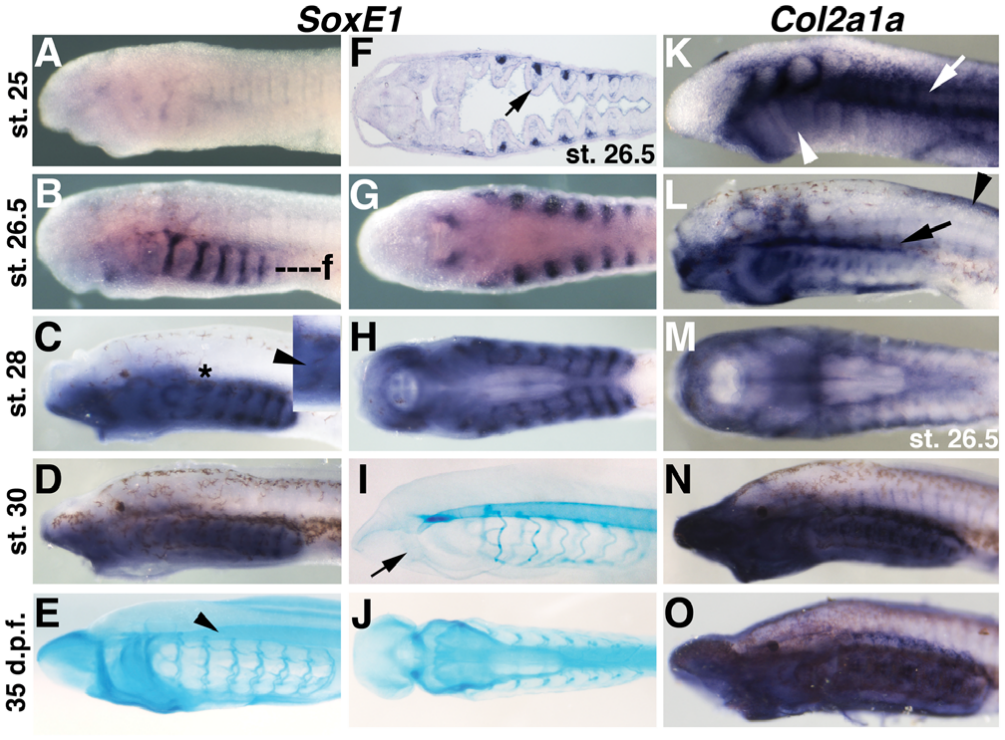
Expression of *SoxE1* and fibrillar collagen, and alcian blue staining during chondrogenesis in lamprey larvae. A) *SoxE1* expression is not observed in post-migratory CNCC at st. 25. B) Strong, specific expression of *SoxE1* in the nascent branchial basket cartilage at st. 26.5. C) Expression of *SoxE1* in the branchial basket cartilage and broadly in the head mesenchyme at st. 28. Compared to st. 26.5, *SoxE1* expression in the branchial bar cartilage is reduced (arrowhead in inset, showing the pharyngeal arch indicated by the asterisk). D) At st. 30, *SoxE1* expression is detectable throughout the head mesenchyme, but has been downregulated in the branchial basket cartilage. E) Alcian blue staining at 35 days post-fertilzation (d.p.f.) in the branchial basket and the mucocartilage of the upper lip, oral region, and ventral pharynx as well as the dorsal fin. Strong staining is also seen in the notochord (arrowhead). F) Frontal section at the level of f in B showing *SoxE1* expression in the nascent branchial basket cartilage and weakly in the CNCC medial to the pharyngeal mesoderm (arrow). G) Ventral view of B showing expression in the mucocartilage around the mouth. H) Ventral view of C. I) Alcian blue staining at st. 30 in the differentiated branchial basket cartilage, notochord, and weakly in the nascent mucocartilage (arrow). J) Ventral view of E showing alcian blue reactivity in the mucocartilage of the ventral pharynx. K) Expression of the lamprey fibrillar collagen gene *Col2a1a* at st. 25. Expression is seen in the somites (arrow) and post-migratory neural crest cells in the pharynx (arrowhead). L) *Col2a1a* expression at st. 26.5 throughout the head mesenchyme including presumptive mucocartilage in the ventral pharynx and around the mouth, and in the presumptive subchordal cartilages(arrow). Minimal expression is seen in the vertical branchial bars. *Col2a1a* mRNA is also present in the dorsal fin (arrowhead) M) Ventral view of L showing mucocartilage expression. N) Broad expression of *Col2a1a* throughout the pharynx and oral region including presumptive mucocartilage and branchial basket cartilage at st. 30. O) At 35 d.p.f. *Col2a1a* is still broadly expressed the head skeleton.

In gnathostomes, the *SoxE* transcription factor, *Sox9*, is a marker of CNCC and pre-chondrocytes [56]. Gnathostome *Col2a1* is a molecular marker for nascent and differentiated cellular cartilage that is directly regulated by *Sox9* [26], [57], [58], [59]. Alcian blue binds the chondroitin sulfate proteoglycans (CSPGs, i.e. Aggrecan) secreted by differentiated cellular cartilage, and is considered diagnostic for this tissue [60]. Together, the combined expression of these molecules is used to monitor the development of cellular cartilage in gnathostomes. To obtain a more complete view of lamprey cartilage development, we examined *SoxE* expression, type A fibrillar collagen expression, and alcian blue reactivity at several time points during chondrogenesis in lamprey (Figure 1).

Previous reports have shown strong early expression of the lamprey *SoxE* paralogs, *SoxE1*, *SoxE2*, and *SoxE3* in premigratory and early migrating neural crest (Tahara st. 21–23) [61] and expression in the nascent branchial basket (st. 26)[38], [39], [40], [47]. However, expression during chondrogenesis (st. 26.5–30+), and in early post-migratory CNCC (st. 25), has not been reported.

We observed a lack of *SoxE1* expression at st. 25, and strong expression in the forming branchial bars at st. 26.5, indicating that *SoxE1* is temporarily down-regulated sometime between st. 23 and st. 25 and reactivated at st. 26 (Figure 1A–G). At st. 28, expression of *SoxE1* in the branchial basket was reduced (Figure 1C,H), particularly in the middle of the vertical branchial bars where cartilage differentiation is first detectable by alcian blue staining[37]. Also at st. 28, we observed broad staining throughout the pharynx including the mesenchyme of the ventral pharynx and oral region. This staining continued until st. 30, when *SoxE1* expression in the branchial bar cartilage was undetectable (Figure 1D).

The loss of *SoxE1* in the branchial bar chondrocytes at st. 30 was coincident with their differentiation as revealed by alcian blue staining (Figure 1I). At 35 days post fertilization (approximately 13 days after st. 30), alcian blue staining revealed the deposition of CSPGs in the fully differentiated branchial basket as well as in the mucocartilage of the oral region (upper lip, first pharyngeal arch, lower lip) and ventral pharynx (Figure 1E, J).

Expression of lamprey fibrillar collagen in the forming branchial basket at select time points (st. 25, st. 26) has been described [38], [39], [62]. We replicated and expanded upon these studies, looking at *Col2a1a* expression at key stages before, during, and after chondrogenesis. As previously reported, we observed broad *Col2a1a* expression in post-migratory cranial neural crest cells and somitic mesoderm at st. 25 (Figure 1K). While *SoxE1* expression is temporarily downregulated at this stage, cranial neural crest expression of *SoxE1* and *SoxE2* is seen earlier at st. 23 and st. 24, consistent with *Col2a1* regulation by *SoxE* in CNCC as seen in gnathostomes. At st. 26.5, strong *Col2a1a* expression is seen in presumptive mucocartilage in the ventral pharynx, upper and lower lips, first pharyngeal arch, and in dorsal fin mesenchyme (Figure 1L,M). Strong *Col2a1a* expression is also observed in a horizontal band of cells dorsal to the pharyngeal arches at the position of the nascent subchordal cartilage bars. Negligible expression was seen in the forming branchial bars at this stage. At st. 28 strong expression of *Col2a1a* was detected throughout the mucocartilage around the mouth, with weaker expression in branchial basket cartilage (Figure 1N). Similar expression was seen later at 35 d.p.f. (Figure 1O).

Our results show *SoxE1* expression is high in post-migratory pharyngeal CNCC, where it is co-expressed with *Col2a1*, and is then downregulated as these cells differentiate into cellular cartilage. This sequence mirrors the down-regulation of *Sox9* seen in differentiating gnathostome cartilages and supports conserved roles for *SoxE* in lamprey branchial basket cartilage and gnathostome cellular cartilage development as previously proposed [40], [47].

In addition to the branchial basket, however, we also observed *SoxE1* expression and alcian blue staining throughout the developing mucocartilage of the oral apparatus and ventral pharynx. Furthermore, we found that these tissues express high levels of fibrillar collagen [38], [39] as originally suggested by Schaffer[51] and Johnels[42]. Due to its staining properties and mesenchymal morphology, mucocartilage is generally considered a derived form of connective tissue unrelated to cellular cartilage[55]. However, co-expression of fibrillar collagen, *SoxE*, and CSPGs in mucocartilage suggest some developmental and/or evolutionary relationship between mucocartilage and cellular cartilage. Hardisty[44] proposed that mucocartilage may represent an undifferentiated embryonic tissue that is carried over into the larval stage. An alternate hypothesis is that mucocartilage represents an evolutionary precursor to bona fide cellular cartilage. We decided to further investigate these possibilities by looking at the expression of three other key regulators of vertebrate chondrogenesis; *Runx*, *Barx*, and *Alx/Cart1*, in the lamprey head skeleton.

### *Runx* genes are expressed in lamprey mucocartilage, but not in the branchial basket cartilage

*Runx* genes are key regulators of both cartilage and bone development in jawed vertebrates. During embryogenesis all three gnathostome *Runx* paralogs, *Runx1,2,3* [18] are expressed in presumptive chondrocytes, and *Runx2* and *Runx3* have been shown to be required for their differentiation into cellular cartilage[10], [20], [21]. After chondrogenesis, *Runx* genes regulate the formation of replacement cartilage and the ossification of endochondral bone[63]. Functional studies suggest *Runx* genes are regulated by both *Sox9* and *Barx* genes and are downstream of these factors in the cartilage GRN[9], [64].

**Figure 2:**
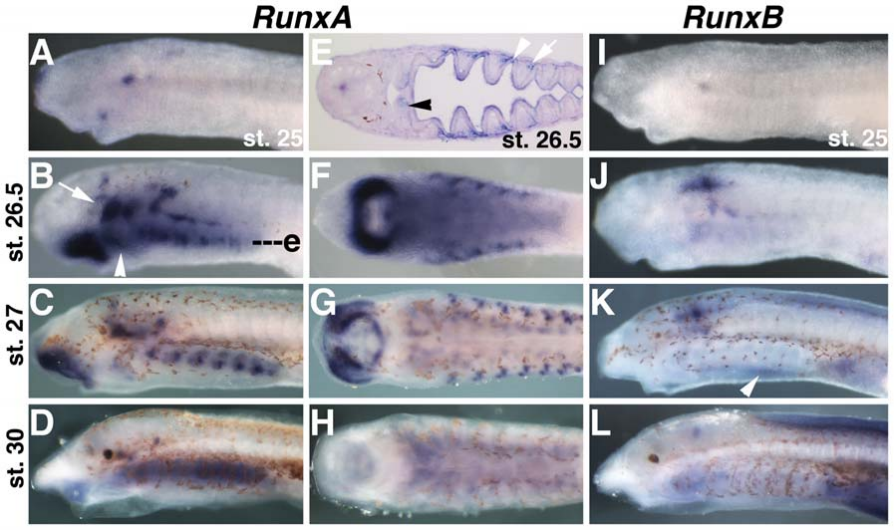
Expression of *RunxA* and *RunxB* in lamprey larvae. A) Localized expression of *RunxA* at st. 25 in oral mesenchyme and nascent cranial ganglia. B, C) Expression of *RunxA* in cranial ganglia (arrow), the intermediate first arch mesenchyme (arrowhead), pharyngeal endoderm, pharyngeal mesoderm, and oral mesenchyme at st. 26.5 and st. 27. D) Expression of *RunxA* in pharyngeal endoderm at st. 30. E) Section at the level of e in B showing *RunxA* expression in the pharyngeal pouch endoderm (arrowhead), pharyngeal mesoderm (arrow), and intermediate first arch mesenchyme (black arrowhead). F) Ventral view of B showing strong expression in the mesenchyme flanking the mouth. G) Ventral view of C. H) Ventral view of D. I) A spot of *RunxB* expression is seen in the nascent cranial ganglia at st. 25. J,K) Expression of *RunxB* in the brain and cranial ganglia at st. 26.5 and st. 27. At st. 27, weak expression is also visible around the heart, in the endostyle (arrowhead), and in the dorsal fin. L) At st. 30, strong expression of *RunxB* is seen in the dorsal fin mesenchyme and around the heart, with weaker staining in the brain and pharyngeal endoderm.

We performed an exhaustive search of *P. marinus* pre-assembly genome and identified several contigs with similarity to the four most highly conserved deuterostome *Runx* exons. For three of these exons, we found two distinct aligning lamprey sequences encoding different proteins, suggesting the presence of two lamprey *Runx* genes (Figure S1, S2). While it is possible that the preassembly *P. marinus* genome does not include all *Runx* genes, the level of coverage, and the presence of two *Runx* genes in hagfish, a related agnathan, suggest this is the full complement of lamprey *Runx* genes. These genes were designated *RunxA* and *RunxB. RunxA* expression was first observed at Tahara st. 25 in the presumptive cranial ganglia and mesenchyme flanking the mouth (Figure 2A). By st. 26.5, the onset of chondrogenesis, expression was seen in the pharyngeal endoderm, cranial ganglia, lateral oral mesenchyme, and intermediate first arch (Figure 2B,E,F). Spots of expression were also observed in the mesodermal core of the pharyngeal arches (Figure 2E). Expression in the cranial ganglia, upper lip, first arch, pharyngeal endoderm and pharyngeal mesoderm was still detectable at st. 27, when cartilage is histologically identifiable by alcian blue staining (Figure 2C,G). By st. 30, when most of the branchial basket cartilage has differentiated, *RunxA* was still detectable in the pharyngeal endoderm and cranial ganglia (Figure 2D,H). *RunxB* deployment was much less extensive at early larval stages, marking bilateral spots in the anterior neural tube and cranial ganglia at st. 25 and 26.5 (Figure 2I,J). At st. 27 additional expression was observed in the dorsal fin mesenchyme, around the heart, and in the endostyle (Figure 2K). At st. 30 strong expression was observed in the dorsal fin and around the heart, with reduced expression in the brain (Figure 2L). By 35 d.p.f. weak expression of both *RunxA* an *RunxB* was seen in the pharyngeal endoderm. (Figure S3).

**Figure 3:**
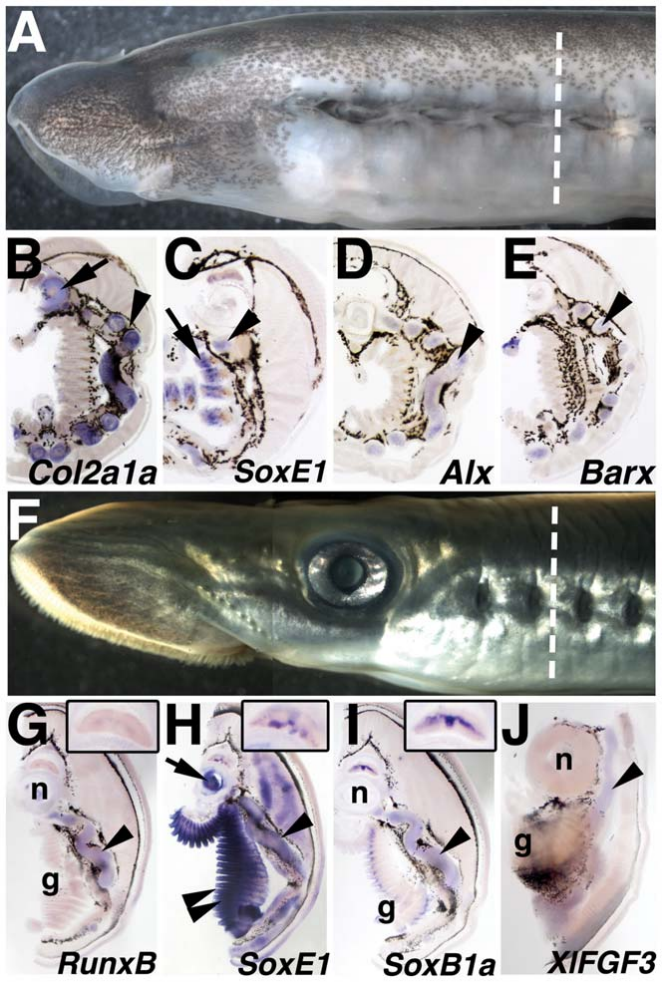
Expression of cartilage GRN components in pre-metamorphic ammocoete larvae and post-metamorphic juvenile lamprey. A) Sections for the *in situ* hybridizations shown in B–E were cut from pre-metamorphic ammocoete larvae through the pharyngeal arches at approximately the level of the dotted line. B) Strong expression of *Col2a1a* is seen above background levels in the notochord (arrow) and in cells in and around the branchial bar cartilage (arrowhead). C) Specific expression of *SoxE1* in the gills (arrowhead). Diffuse staining in the branchial cartilage (arrowhead) is likely due to background as can be seen with two negative control riboprobes corresponding to a lamprey *SoxB1* gene (I, arrowhead) and *Xenopus laevis FGF3* (J, arrowhead). No specific expression of *Alx* (D) or *Barx* (E) was seen in branchial bar cartilages. F) Sections for the *in situ* hybridizations shown in G–J were cut from post-metamorphic juvenile lampreys through the pharyngeal arches at approximately the level of the dotted line. No expression of *RunxB* (G) or *SoxE1* (H) was seen in the branchial basket cartilage (arrowheads) after metamorphosis. In contrast, strong, specific reactivity with the *SoxE1* and *SoxB1a* probes is seen in different subsets of spinal cord neurons (H, I, insets). *SoxE1* mRNA is also detected in the notochord (H, arrow), and gills (H, double arrowhead). I) Control *in situ* hybridization with lamprey *SoxB1*a showing neural expression, and expression in the gills.

We then examined *Runx, SoxE1*, and *Col2a1a* expression in pre-metamorphic ammocoete larvae and recently metamorphosed juvenile lampreys, to determine if these genes are activated in cellular cartilage around the time of metamorphosis. While we did see expression of *Col2a1a* in the branchial bars (Figure 3B), we observed no specific signal in any pharyngeal tissue at these stages with either the *RunxA* (not shown) or *RunxB* riboprobes (Figure 3G). Similarly, *SoxE1* expression above background levels was not seen in pre- or post-metamorphic branchial basket cartilage, though specific expression was seen in the gills, notochord, and a subset of cells in the spinal cord (Figure 3C,H).

Taken together, we detected no lamprey *RunxA* or *RunxB* expression in branchial basket cartilage at any stage, either before or after metamorphosis. While it is formally possible that an unidentified lamprey *Runx* paralog is expressed in lamprey branchial basket cartilage, we view this as unlikely. The apparent lack of *Runx* expression in lamprey cartilage contrasts with gnathostomes where *Sox9* and all three *Runx* genes are broadly co-expressed and interact extensively to regulate chondrogenesis. Assuming *RunxA* and *RunxB* represent the full complement of lamprey *Runx* genes, this difference implies either that *Runx* lost its ancestral function in cartilage development in the lamprey lineage, or that *Runx* genes were not part of the ancestral vertebrate cartilage GRN.

Recent work using qPCR on adult tissue suggests enrichment of *Runx* transcripts in the cartilage of adult hagfish, leading to speculation that *Runx* genes drive skeletogenesis in these agnathans[65]. Hagfish have historically been considered basal jawless craniates, but modern molecular phylogenies support their grouping with lampreys in a single agnathan clade[66]. If *Runx* is in fact required for chondrogenesis in hagfish, it would support the lineage-specific loss of *Runx* from the lamprey cartilage GRN.

Alternately, *Runx* expression in adult hagfish could reflect a function in adult tissue maintenance or metabolism unrelated to cartilage specification during development. In jawed vertebrates, *Runx* genes perform a wide range of functions in embryonic and adult tissues. Gnathostome *Runx* genes are downregulated in differentiated adult cartilage and upregulated in a variety of other tissues including skin, liver, intestine, thyroid and blood where they regulate cell division and stem cell quiescence [19], [67], [68], [69]. Consistent with a general role in adult tissue maintenance, hagfish *Runx* transcripts were found in every hagfish tissue examined, with slightly higher levels in hard cartilage[65]. In either case, the lack of detectable enrichment of lamprey *Runx* mRNA in branchial basket cartilage indicates *Runx* genes are dispensable for the development of histologically discernable cellular cartilage. We propose this reflects the basal vertebrate state, but it could formally represent the presence of a derived chondrogenic GRN specific to lampreys. *Runx* expression during cartilage development in hagfish embryos would help distinguish between these two scenarios.

While lamprey *Runx* expression was not observed in the definitive cellular cartilage of the branchial basket, strong, localized *RunxA* expression was observed in subpopulations of nascent mucocartilage around the mouth and in the intermediate portion of the first arch. This restricted expression suggests lamprey *Runx* genes may be performing tissue-specific functions in the development of particular kinds of mucocartilage, rather than acting as general drivers of skeletogenesis as they are in gnathostomes.

Other domains of *Runx* expression included the brain, cranial ganglia, and pharyngeal endoderm in early larvae (st. 25–26.5) and the heart, and dorsal fin mesenchyme in late larvae (st. 27–30). Expression of *Runx* genes is observed in the cranial ganglia of all gnathostomes examined, indicating a deeply conserved role for *Runx* in the development of these structures[18], [21]. Expression in the dorsal fin may be related to a skeletogenic function as these cells also express *SoxD* and fibrillar collagen (Figure 1)[38], and react with with alcian blue (Figure 1E).

An endodermal specification function for *Runx* genes appears to be evolutionarily ancient as gnathostomes, amphioxus, sea urchin, and *C. elegans* all express *Runx* in the nascent gut, and *Runx* is required for gut formation in *C. elegans* [7], [67], [70]. *Runx* expression in the pharyngeal endoderm of adult amphioxus has been interpreted as evidence of a rudimentary skeletogenic program in this tissue[65]. Given the pleiotropic nature of *Runx* function, and the pan-metazoan expression of *Runx* genes in endoderm, it seems equally likely that *Runx* genes are performing some other function in this tissue.

### Lamprey *Barx* is expressed in lower lip mucocartilage and CNCC in the medial pharyngeal arches, but not in developing branchial bar cartilage

Recent work has shown that *Barx* is required for the differentiation of CNCC-derived cartilage in the zebrafish head where it is downstream of FGF signaling and regulates *Runx* expression[9]. Similar broad expression of *Barx* in the CNCC of chick and mouse suggest this role is conserved among all gnathostomes[17], [71]. In addition, functional studies in mouse have demonstrated an essential role for *Barx* in the chondrogenesis of the mesoderm-derived appendicular skeleton[27].

**Figure 4:**
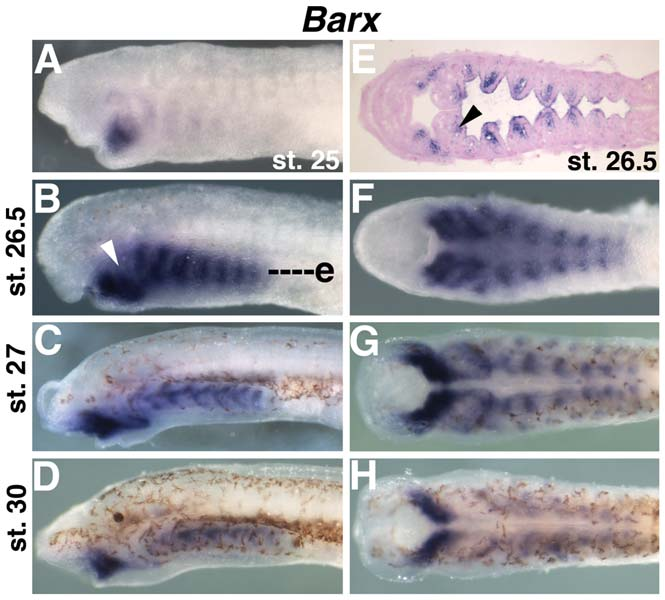
Expression of *Barx* in lamprey larvae. A) *Barx* transcripts at st. 25 in the mesenchyme of the lower lip. B–D) *Barx* expression at st. 26.5, st. 27, and st. 30 in the lower lip (ventral first arch), intermediate first arch (arrowhead), and in the posterior pharyngeal arches. E) Frontal section at the level of e in B reveals *Barx* expression in the pharyngeal arches is restricted to the CNCC medial to the pharyngeal mesoderm. Barx transcripts are also detected in the mesenchyme of the lower lip and the intermediate first arch (arrowhead). F–H) Ventral views of B, C, and D, showing expression in the mucocartilage of the lower lip and in the medial aspect of the pharyngeal arches.

We isolated a lamprey *Barx* ortholog, and described its expression in the first pharyngeal arch at st. 26.5 in a previous study[72]. Here we detail lamprey *Barx* expression from embryonic through late larval stages in all tissues. *Barx* transcripts are first weakly detectable in a stream of CNCCs migrating into the region of the first pharyngeal arch at st. 24 (data not shown). At st. 25, *Barx* expression is seen in the lower lip and the forming pharyngeal arches (Figure 4A). At st. 26.5 and 27, strong *Barx* expression is seen in the lower lip, extending dorsally into the intermediate region of the first pharyngeal arch as previously described (Figure 4B,F,C,G). Expression is also seen in presumptive CNCC-derived mesenchyme positioned medial to the pharyngeal arch mesoderm in the posterior arches (Figure 4E). These domains of expression persist into st. 30 when chondrogenesis of the branchial bars is complete (Figure 4D,H). Similar expression is still apparent at 35 d.p.f (Figure S3). No *Barx* expression was seen in the pharynx of pre-metamorphic ammocoete larvae (Figure 3E) or post-metamorphic juveniles (not shown).

As with *Runx*, we noted an absence of *Barx* expression in the laterally-positioned pharyngeal CNCCs which give rise to the definitive cellular cartilage of the gill bars. Thus, like *Runx*, *Barx* is likely not directly involved in the development of lamprey cellular cartilage, whereas it is required for definitive cartilage formation in gnathostomes.

Although *Barx* expression did not mark CNCC in the lateral pharynx, we did observe *Barx* expression in CNCC medial to the mesodermal core of each pharyngeal arch. These cells express other markers of CNCC including *tfap2*, *Dlx,* and *SoxE*, as well as fibrillar collagen[39], [72]. However, they do not react with alcian blue or correspond to histologically recognizable mucocartilage. In lamprey, this region of the pharyngeal arches gives rise to the smooth muscle of the branchial veins and arteries [43], tissue which is CNCC-derived in gnathostomes. We speculate that lamprey *Barx* expression in the medial portion of the pharyngeal arches marks CNCC fated to form the branchial vasculature.

### Lamprey *Alx* marks upper lip mesenchyme and portions of the branchial basket cartilage

Gnathostomes possess three *Alx* paralogs, *Alx3*, *Alx4*, and *Cart1*. The expression and function of these factors during cartilage development have been studied almost exclusively in mouse, where all three paralogs mark chondrogenic mesenchyme in the frontonasal mass, pharyngeal arches, and limb buds[25], [73]. Consistent with their expression patterns, double knock-outs of *Alx3* and *Alx4* result in severe reductions in the craniofacial and appendicular skeletons[22]. Among invertebrate deuterostomes, *Alx* is required for skeletal development in sea urchin[74], and amphioxus *Alx* is expressed in pharyngeal mesoderm thought to secrete acellular cartilage[7], suggesting an ancient role for *Alx* genes in skeletogenesis.

**Figure 5:**
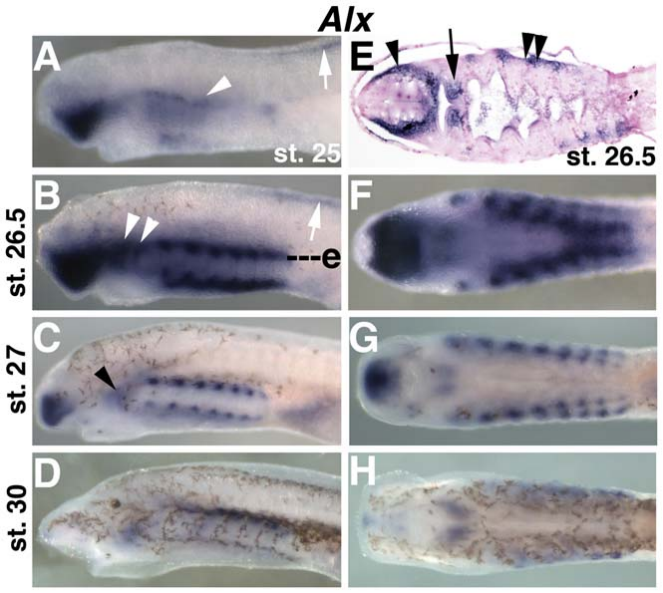
Expression of *Alx* in lamprey larvae. A) Strong *Alx* expression in the upper lip at st. 25, with weaker expression in the dorsal (arrowhead) and ventral aspects of the forming pharyngeal arches. Expression is also seen in the forming dorsal fin (arrow). B) Expression of *Alx* in upper lip and strongly in the dorsal and ventral aspects of the posterior (3^rd^–8^th^) pharyngeal arches. In the first and second arches, *Alx* expression is only seen dorsally (arrowheads). Expression is also seen in the dorsal fin (arrow). C) At st. 27, expression in the dorsal and ventral aspects of the pharyngeal arches, upper lip, and dorsal first arch (arrowhead). Expression in the dorsal fin has been lost. D) At st. 30, *Alx* expression is seen in the dorsal first arch and weakly in the subchordal chondrocytes. E) A frontal section at the level of e in B showing *Alx* expression in CNCC-derived upper lip mesenchyme surrounding the anteriormost neural tube (arrowhead). Expression is also seen in the dorsal portions of the first arch (arrow) and the posterior arches (double arrowheads). F) Ventral view of B showing expression in the upper lip and the nascent hypobranchial chondrocytes. G) Ventral view of C. H) Ventral view of D.

We found multiple contigs corresponding to a single *Alx* gene in the *P. marinus* genome (Figure S1, S2), suggesting it is the only lamprey *Alx* homolog. We then examined its expression in embryos and larvae during cartilage development. Lamprey *Alx* expression was first seen at st. 23.5 in a spot of mesenchyme in the forming upper lip (data not shown). This expression expanded and intensified to fill the mesenchyme of the upper lip at st. 25 (Figure 5A). Additional expression was seen in the dorsal and ventral aspects of the forming pharyngeal arches and in the forming dorsal fin. By st. 26.5, expression was seen in the mesenchyme of the dorsal and ventral pharyngeal arches, upper lip, and dorsal fin (Figure 5B,F). Expression was excluded from the lower lip mesenchyme and the intermediate portion of the pharyngeal arches. This expression resolved into distinct dorsal and ventral spots in each arch at st. 27 (Figure 5C,G). By st. 30, *Alx* expression in the pharynx had diminished, but was still detectable in the first arch and subchordal chondrocytes (Figure 5D,H). This expression pattern persisted until 35 d.p.f. (Figure S3), though no *Alx* expression was detectable in the pharynx of ammocoetes (Figure 3D) or metamorphosed juveniles (not shown).

In gnathostomes, *SoxE* and *Alx* genes are co-expressed in CNCC-derived chondrogenic mesenchyme[25]. While no regulatory relationship between the two factors has been demonstrated, *Sox9* and *Alx*3/4 double mutant mice have similar hypoplastic skeletal phenotypes, suggesting they operate within the same cartilage GRN[22], [75]. Unlike their gnathostome counterparts, lamprey *SoxE* and *Alx* genes are not broadly co-expressed in nascent cellular cartilage. While lamprey *SoxE* marks the entire branchial basket cartilage, lamprey *Alx* is restricted to the dorsal and ventral aspects, and is excluded from the vertical branchial bar cartilage. Recent work has shown that the lamprey branchial basket consists of morphologically and molecularly distinct cell types arranged along its dorso-ventral (DV) axis[37], [72]. In the central portion of the pharyngeal gill bars, vertically stacked branchial bar chondrocytes display a highly ordered discoidal “stack-of-coins” morphology. Dorsally, subchordal, parachordal and trabecular chondrocytes are disorganized and polygonal in shape. Ventrally, the horizontal hypobranchial bars have a semi-ordered pseudo-discoidal morphology. *Alx* expression in the pharynx corresponds to the forming subchordal and hypobranchial chondrocytes. Both subchordal and hypobranchial chondrocytes likely serve as rigid structural elements, in contrast to the central discoidal chondrocytes, which form flexible bars that bend and recoil during pharyngeal pumping. It is possible that *Alx* expression in the lamprey branchial basket identifies a particular kind of rigid structural cartilage homologous to the cellular cartilage that comprises the bulk of the gnathostome head skeleton.

Similarly, *Alx* expression in the upper lip mesenchyme suggests *Alx* may also specify a type of mucocartilage. Historical descriptions classify the skeletal tissue in the upper lip, lower lip, first pharyngeal arch, and ventral pharynx as a single kind of generic mucocartilage[42], [43], [51]. However, at the stages we examined, the skeletal tissue around the larval lamprey mouth appears compact, while the presumptive mucocartilage of the ventral pharynx forms a loose mesenchyme. *Alx* expression in the upper lip may confer some unique physical properties that distinguish this skeletal tissue from the loose mucocartilage in the ventral pharynx.

Like *Runx*, and *Col2a1a*, we also observed *Alx* transcripts in the dorsal fin mesenchyme, a tissue that reacts with alcian blue (Figure 1) and is at least partially neural crest-derived [45]. Coexpression of *Runx* and *Alx* is not seen in any skeletal tissue in the head, suggesting lamprey dorsal fin mesenchyme may represent a mucocartilage-like tissue unique to the trunk.

### Summary of *SoxE1, Col2a1a, CSPG, Runx, Barx,* and *Alx* expression in lamprey skeletal tissues

**Figure 6:**
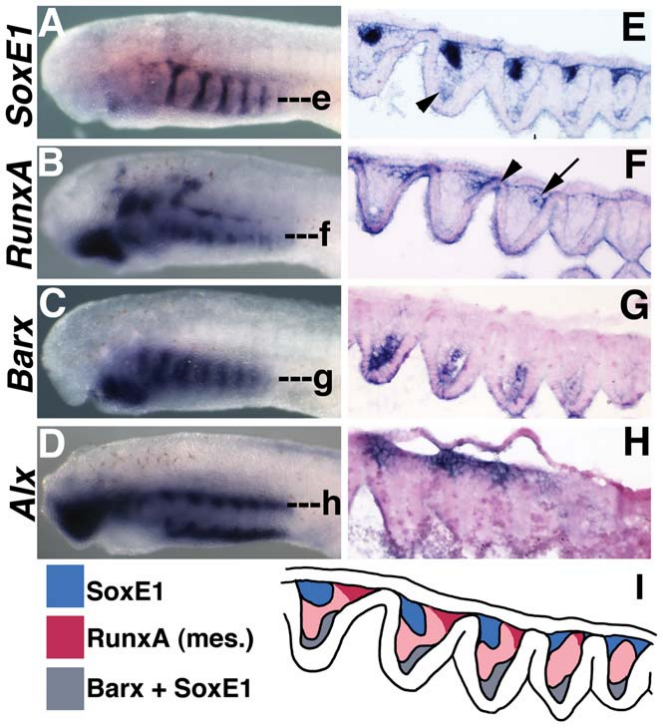
Comparison of lamprey *SoxE1*, *RunxA*, *Barx*, and *Alx* expression in the pharyngeal arches at st. 26.5. A–D)*SoxE1*, *RunxA*, *Barx*, and *Alx* expression at st. 26.5 in wholemount *in situ*-hybridized larvae. While the gnathostome homologs of *SoxE1*, *RunxA*, *Barx* and *Alx* are all coexpressed in developing cellular cartilage, these genes have largely non-overlapping expression patterns in lamprey. Of the 4 genes, only *SoxE1* marks the stack-of-coins cartilage of the vertical branchial bars. *Alx* marks the subchordal and hypobranchial chondrocytes as well as the mucocartilage of the upper lip. *Barx* and *Runx* are not expressed in lamprey cellular cartilage, but mark different populations of mucocartilage around the mouth. E–G) Expression of *SoxE1*, *RunxA*, and *Barx* in the pharyngeal arches at st. 26.5 as revealed by frontal sections through the middle of the branchial bars. E) Only *SoxE1* marks the nascent branchial bar cartilage. F) *RunxA* is expressed in pharyngeal mesoderm (arrow) and endoderm (arrowhead). G) *Barx* marks mesenchyme medial to the mesoderm. This mesenchyme also weakly expresses *SoxE1* (arrowhead in E). H) Frontal section through h in D showing *Alx* expression in CNCC cells which will give rise to subchordal chondrocytes. I) Summary of *SoxE1*, *RunxA* and *Barx* expression in the pharyngeal arches at the level of the branchial bars.

**Figure 7:**
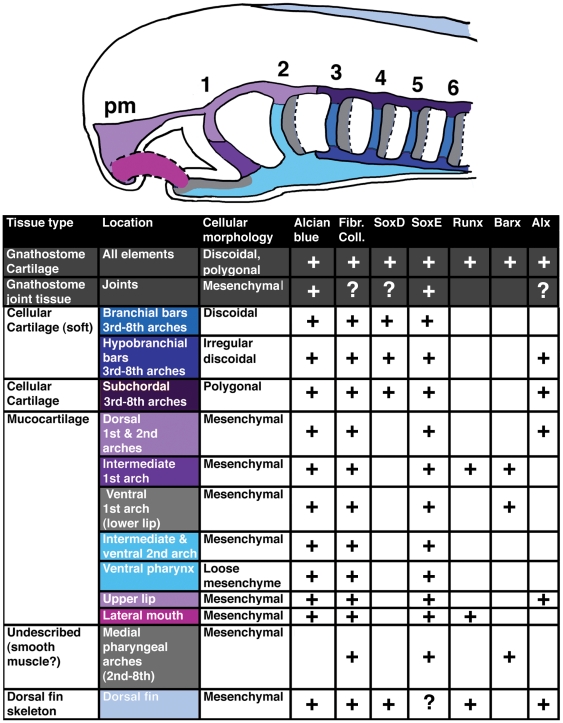
The diversity of skeletal tissues in the larval lamprey head. Based on morphology and gene expression, we speculate that lamprey larvae may possess as many as 11 distinct types of skeletal tissue. This includes 3 kinds of cellular cartilage, 7 kinds of mucocartilage, and a mucocartilage-like skeletal tissue unique to the dorsal fin. The diagram shows the first 6 pharyngeal arches. The upper lip mesenchyme is derived from the premandibular cranial neural crest (pm). For reference, cellular morphology and gene expression in gnathostome cellular cartilage and joint tissue are shown in the shaded rows.

In sum, our results show expression of *SoxE1, Col2a1a,* and staining with alcian blue (a proxy for CSPG expression) in all mucocartilage and cellular cartilage of the lamprey head at early larval stages (st. 26.5–st. 30). During this period, *RunxA* expression was seen in a small subpopulation of mucocartilage flanking the mouth, while *RunxB* was observed in dorsal fin mesenchyme. *Barx* expression was observed in a portion of the mucocartilage in the lower lip/first arch, while *Alx* expression was detected in upper lip mucocartilage, two subpopulations of cellular cartilage, and dorsal fin mesenchyme. No significant overlap of *RunxA*, *RunxB*, *Barx* or *Alx* expression was seen cellular cartilage (Figure 6), or any other skeletal tissue except in the intermediate first arch (*Barx* and *Runx*) the dorsal fin mesenchyme (*RunxB* and *Alx*) (Figure 7). However, *RunxA*, *Barx* and *Alx* were all expressed with *SoxE1* and *Col2a1a* in various skeletal tissues and at some point between st. 26.5 and st. 30. In pre-metamorphic ammocoete larvae and post-metamorphic juvenile lampreys, only expression of *Col2a1a* was detectable above background levels in branchial basket cartilage.

### A hypothetical scenario for the origin and evolution of vertebrate cartilage

We are taking a molecular approach to examine possible evolutionary relationships between invertebrate skeletal tissue, gnathostome cartilage, and the skeletal tissues of lamprey. Our data show that, despite similarities with gnathostome cellular cartilage, the cellular cartilage of the lamprey branchial basket deploys a rudimentary differentiation program that does not incorporate *Runx* or *Barx*. Furthermore, we find that the flexible “stack of coins” chondrocytes of the branchial basket do not express *Alx* genes and are thus molecularly distinct from the rigid polygonal subchordal and hypobranchial chondrocytes (Figure 6). Finally, we find that lamprey mucocartilages, though morphologically very different from gnathostome cellular cartilage, express various combinations of genes involved in gnathostome chondrogenesis (Figures 6, 7).

Taken together, our data reveal an array of histologically and molecularly distinct skeletal tissue types in the larval lamprey head, each displaying a subset of the features seen in gnathostome cellular cartilage (Figure 7). This contrasts with the gnathostome condition in which all embryonic cellular cartilage appears to deploy a single tightly conserved chondrogenic GRN. Assuming that the lamprey head skeleton approximates the basal vertebrate condition, our results have two significant evolutionary implications. First, they suggest that the evolution of the gnathostome head skeleton involved a marked reduction in skeletal tissue diversity. This implies that the basal vertebrate state is the possession of multiple cartilage-like tissue types, while gnathostomes display a derived dependence on a single kind of cellular cartilage. Second, our results suggest that this loss of skeletal tissue diversity was coincident with the consolidation of several rudimentary skeletogenic GRNs into a single chondrogenic GRN. Thus, during gnathostome evolution, gene programs operating primitively in different types of skeletal tissue became co-expressed in a single type of cellular cartilage.

**Figure 8:**
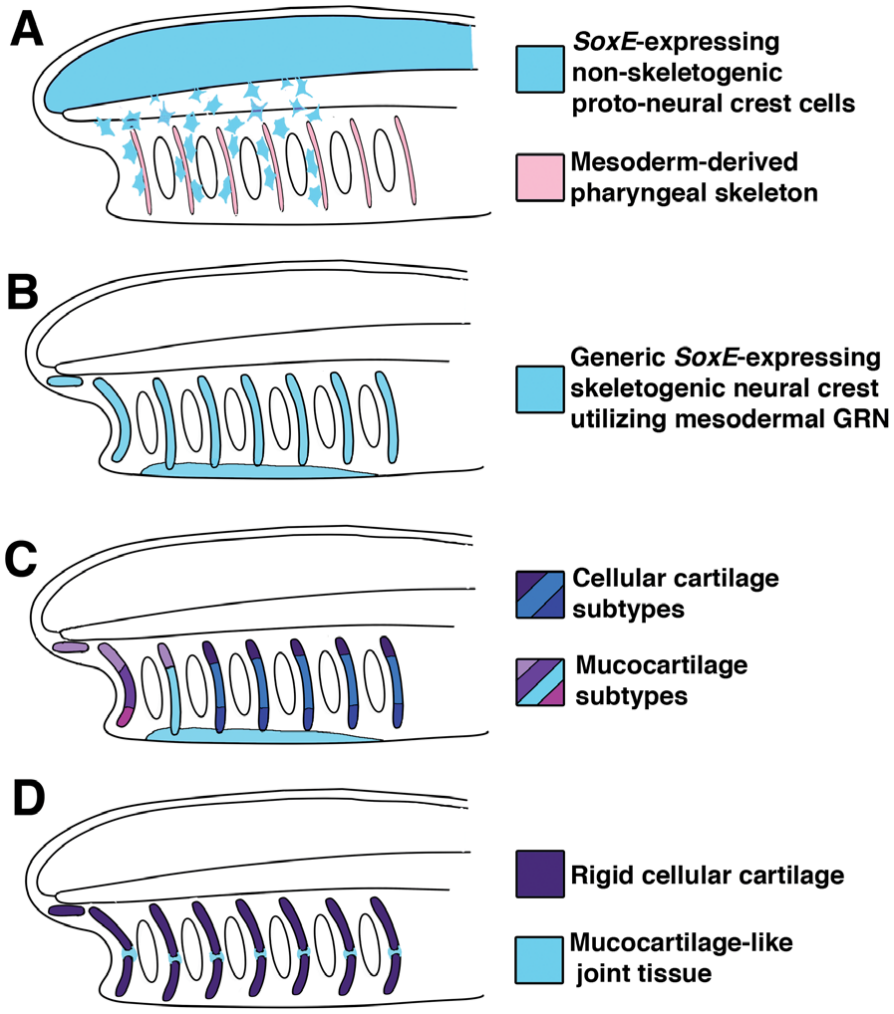
A hypothetical scenario for the origin and evolution of vertebrate cartilage. A) Based on the expression of *SoxE* genes throughout the neural tube of amphioxus larvae, and the presence of migratory neural cells in urochordates and other invertebrates, we posit that the ancestral pre-vertebrate chordate possessed migratory neural tube cells that expressed *SoxE* genes (light blue). These cells migrated into the pharynx and generated neural and/or pigment cells, but were not skeletogenic. The pharyngeal skeleton at this stage (pink) resembled that of amphioxus, consisting of acellular cartilage incorporating acid mucopolysaccharides and secreted by pharyngeal mesoderm expressing *Twist, Ets, Id, Alx, CSPGs* and fibrillar collagen. B) The origin of bona fide skeletogenic neural crest, and the first craniates, was marked by the activation of this mesodermal skeletogenic gene program in *SoxE*-expressing migratory neural tube cells (light blue). This ancestral form of cellular cartilage provided structural support for the evolving head. C) In the lineage leading to the common ancestor of modern jawed and jawless vertebrates, this generic skeletal tissue diversified into several subtypes with distinct molecular and structural properties via cooption of *Runx* and *Barx* and the selective loss of *Alx* expression. This included definitive “cellular cartilage” similar to that seen in gnathostomes and multiple mucocartilage subtypes as seen in lamprey. This basal condition is maintained in lamprey. D) In gnathostomes this heterogenous head skeleton was replaced with a skeleton consisting mainly of a single type of rigid cellular cartilage. Coincident with this shift was the consolidation of *SoxE1*, *Runx*, *Barx*, and *Alx* genes into a single gnathostome cellular cartilage gene network. Based on similar gene expression and cell morphology, the joint tissue seen in gnathostomes (light blue) may represent a basal form of skeletal tissue similar to mucocartilage in the ventral pharynx of lamprey.

We previously proposed that genetic cooption of mesodermal gene networks by the evolving cranial neural crest drove the origin of vertebrate cellular cartilage[7]. Our current results suggest similar cooption events occurred during the evolution of gnathostome cellular cartilage. Below we integrate our current and previous data and propose a stepwise mechanistic scenario for the origin and evolution of vertebrate cellular cartilage (Figure 8).

### Step 1: The origin of skeletogenic CNCC

Our current results, and previous work, reveal a set of core features shared by all skeletogenic neural crest cells, including gnathostome cellular cartilage, lamprey cellular cartilage, and lamprey mucocartilage. These include; 1) differentiation from a mesenchymal precursor cell, 2) persistent expression of *Twist, Ets, Id,* and *SoxE* after migration into the pharynx[39], [47], 3) secretion of CSPGs[37], and 4) expression of fibrillar collagen. Notably, this list does not include expression of *Runx* or *Barx,* which our data show are not general markers of neural crest-derived skeletal tissue.

Using these criteria as a minimal definition of vertebrate neural crest-derived skeletal tissue, we can then ask if any protochordate tissue possesses all, or most, of these features. As has been shown previously, *Twist, Ets, Id, Alx, and* fibrillar collagen are coexpressed in the pharyngeal mesoderm of amphioxus larvae[7], [50]. Furthermore, this tissue displays mesenchyme-like cell morphology and may give rise to acellular cartilage bars composed of CSPGs[7], [50]. Thus, skeletogenic mesoderm with most attributes of skeletogenic neural crest cells, but lacking *SoxE* expression, was likely present in the first chordates.

The existence of migratory neuroblasts in a range of invertebrates [76], [77], [78] suggests that the vertebrate ancestor also possessed some kind of migratory neural tube cell. Assuming that co-expression of *Twist, Ets, Id, Alx, and* fibrillar collagen reflects a conserved GRN, it is possible that skeletogenic neural crest cells evolved via activation of this GRN in non-skeletogenic migratory neural tube cells. The lack of *SoxE* expression in amphioxus pharyngeal mesoderm further suggests this cooption involved the integration of *SoxE* into the evolving skeletogenic GRN. How these changes occurred is unclear. Aside from their function in skeletogenesis, vertebrate *SoxE* paralogs are required for the specification and differentiation of all neural crest cell lineages, as well as for the development of glia and the otic placode[79]. In addition, amphioxus *SoxE* is expressed throughout the nascent nervous system in early larvae, but not in any obviously skeletogenic tissues aside from a few cells in the notochord [80]. These data imply that the ancestral function of chordate *SoxE* was in neural development, rather than skeletogenesis, as has been proposed [65], [81]. Furthermore, these results suggest that migratory neural tube cells could have acquired *SoxE* simply by maintaining *SoxE* expression after leaving the CNS. Based on these observations, we propose that skeletogenic neural crest cells evolved by activation of a mesodermal skeletogenic GRN in migratory neural tube cells expressing *SoxE*. (Figure 8A,B). A role for *SoxE* genes in skeletogenesis then evolved later in the vertebrate lineage as *SoxE* was integrated into this skeletogenic GRN.

The cartilage GRN in vertebrates is induced and maintained by signals secreted by the pharyngeal endoderm and ectoderm, including *Hedgehogs* and *FGFs* [82], [83]. Provocatively, *FGF* and *Hedgehog* ligands are expressed in the amphioxus pharynx at larval stages when skeletogenesis is likely initiated[7], [84]. We further speculate that the activation of mesodermal skeletogenic gene programs could have evolved as proto-CNCC cells gained responsiveness to these signals.

Based on *Runx* and *SoxE* co-expression in the foregut of adult amphioxus, it has been proposed that cooption of a endodermal skeletogenic GRN(s) incorporating these factors drove the appearance of skeletogenic CNCC[65], [81]. However, expression of lamprey *SoxE1* and *Runx* in non-skeletogenic pharyngeal endoderm and gills (Figure 2, Figure 3B) suggests these factors perform non-skeletogenic functions in chordate endoderm. Furthermore, our data showing that *Runx* is not expressed in lamprey cellular cartilage implies it is not a core feature of chordate skeletal development. Finally, the early role of *SoxE* genes in the initial formation of all neural crest cells, suggests its ancestral function was in neural crest cell specification rather than chondrogenesis[79]. Thus, cooption of *Runx* and *SoxE* from endoderm was likely not involved in the origin skeletogenic neural crest cells. Instead, our data support later recruitment of *Runx* by *SoxE*-expressing skeletogenic neural crest cells in the gnathostome lineage (see below).

### Step 2: The diversification of CNCC-derived skeletal tissues in early jawless vertebrates

The “New Head” hypothesis suggests that the original function of vertebrate cartilage was to provide structural support for pharyngeal pumping[1]. The concurrent expansion of the CNS, anterior sense organs, and cranial ganglia also likely required additional skeletal support, as did the novel structural demands on the oral region of early vertebrate predators. In the first vertebrates, we posit that these various functions were performed by a generic CNCC-derived skeletal tissue expressing *Twist, Id, Ets*, *Alx*, *SoxE*, and fibrillar collagen, and secreting CSPGs (Figure 8B). Based on gene expression in lamprey, we further speculate that this generic CNCC-derived head skeleton then diversified, with different regions taking on specialized properties in response to particular structural demands. During this phase of vertebrate skeletal evolution, *Runx*, and *Barx*, were recruited to different parts of the CNCC-derived skeleton, and *Alx*, which was initially expressed in all skeletogenic CNCC, was lost from some regions. These changes in gene expression drove the establishment of several different skeletal tissue types with distinct physical properties (Figure 8C). Among these were compact tissues composed of cells with polygonal and discoidal morphologies that would be histologically identifiable as proper “cellular cartilage”, and several types of mesenchymal skeletal tissues in the head and dorsal fin. This primitive diversity of CNCC-derived skeletal cell types is thus preserved in lamprey, where *Runx*, *Barx*, and *Alx* genes mark different subsets of cartilage and mucocartilage around the mouth and in the pharynx (Figure 7). Localized expression of these factors would also require some level of underlying anteroposterior (AP) and dorsovental (DV) patterning in the oro-pharyngeal region. Consistent with this, recent work suggests sophisticated gnathostome-like AP and DV patterning systems were in place before the divergence of jawed and jawless vertebrates[72], [85].

### Step 3: The evolution of gnathostome cartilage

While chondrogenic gene expression in lamprey identifies several molecularly distinct skeletal tissues (Figure 7), the ubiquitous coexpression of chrondrogenic GRN components in the gnathostome head skeleton suggests the presence of only one major cartilage type. If lamprey represents the basal state, then the evolution of the gnathostome head skeleton was associated with a reduction in skeletal tissue diversity. A hallmark of the lamprey head skeleton is the preponderance of soft, flexible skeletal tissues such as the mucocartilage of the oral region and ventral pharynx, and the discoidal “soft” cartilage of the branchial basket. This contrasts with the gnathostome condition, where uniformly rigid cartilage elements articulate at foci of soft joint tissue. Rigid “hard” cartilages have been described in lamprey based on their staining properties[42], [43], [51]. Interestingly, these cartilages have the polygonal morphology typical of most gnathostome cartilages and express *Alx*. We speculate that a similar type of specialized, polygonal, *Alx*-positive cartilage was present in the first vertebrates where it constituted a small portion of the head skeleton. In the gnathostome lineage, this rigid cartilage proliferated, becoming the dominant component of the head skeleton (Figure 8D). The broad expression of *Runx* and *Barx* in the gnathostome head skeleton suggests this transition also involved the expanded expression of these factors. We posit that *Runx* and *Barx* may have initially conferred novel structural properties upon this tissue. The transition to rigid structural cartilage may have been driven by the novel physical requirements of holding and processing increasingly large prey items and supporting and protecting a large CNS and sense organs. Similar selective pressures may have driven the subsequent evolution of bone.

In addition to rigid cellular cartilage, gnathostomes also possess soft joint tissue between cartilaginous elements in the head. Like cellular cartilage, these cells are CNCC-derived and initially express *SoxE*, *Twist, Ets*, and *Id* and stain with alcian blue [86]. However, unlike, cellular cartilage, this tissue remains mesenchymal and does not express *Barx* and *Runx* [9], [60]. Based on morphology and *Gdf5/6/7* expression we previously proposed an evolutionary relationship between gnathostome joint tissue and lamprey mucocartilage in the ventral pharynx[72]. Our current results showing that mucocartilage in the ventral pharynx does not express *Runx* and *Barx* lend support this hypothesis. We speculate that gnathostome joints may have evolved by the redeployment of a primitive mucocartilage-like skeletal tissue to the regions between rigid cellular cartilage condensations (Figure 8D).

## Materials and Methods

### Ethics Statement

All methods were reviewed and approved by the University of Colorado, Boulder IACUC protocol 08-07-MED-02.

We performed an exhaustive search of the 5.9X coverage, preassembly *Petromyzon marinus* genome for homologs of gnathostome *Runx*, *Barx* and *Alx* genes by repeated BLAST[87] searching with gnathostome, amphioxus, and sea urchin protein sequences. We then designed exact-match PCR primers for the two *Runx* paralogs (*RunxA* and *RunxB*), and the single *Barx* and *Alx* genes. *Runx and Barx* exons were PCR amplified from adult lamprey genomic DNA according to standard methods. *Alx* was isolated from embryonic cDNA using the GeneRacer kit (Invitrogen). A *SoxE1* fragment corresponding to nucleotides 211-619 of the published *SoxE1* nucleotide sequence[47] was amplified from embryonic cDNA for use as a riboprobe template.

Embryos and early larvae for *in situ* hybridization were obtained from adult spawning phase sea lampreys (*Petromyzon marinus*) as previously described[88]. 5–10 cm pre-metamorphic ammocoete larvae, and newly metamorphosed juvenile lamprey were collected from streams feeding Lake Huron in the Fall with a backpack electroshocker and kept in chilled holding tanks until fixed in MEMFA (MOPS buffer, EGTA, MgSO_4_, Formaldehyde). Thick cross sections of 100–200 microns were then cut through the pharynx using a vibratome.

*In situ* hybridization on embryos, larvae, and vibratome sections of juveniles, was performed with 300–500 bp riboprobes against coding regions and/or 5′ untranslated regions using a high-stringency hybridization protocol [49], [89]. Key parameters of this protocol include post-hybridization washes at 70°C, and the use of a low salt, low pH hybridization buffer (50% formamide; 1.3X SSC, pH 5.0; 5 mM EDTA, pH 8.0; 50 µg/ml tRNA; 0.2% Tween-20; 0.5% CHAPS; 100 µg/ml heparin). *In situ-*hybridized embryos were then cryostat sectioned and counterstained using Nuclear Fast Red (Vector Laboratories, Burlingame, CA). Alcian blue staining was as previously described [37] with the addition of a 2 hour bleaching step in 1%H_2_O_2_, 5% formaminde, and.5% SSC on a fluorescent light box.

## Supporting Information

Figure S1 **Phylogenetic analysis of lamprey** ***Runx*** **and** ***Alx*** **genes.** Lamprey *RunxA* and *RunxB* group with deuterostome *Runx/Runt* homologs with high confidence values using either the Neighbor-Joining (A) or Maximum Likelihood (B) methods. Lamprey *Alx* groups with deuterostome *Alx/Cart* homologs with high confidence values using either the Neighbor-Joining (C) or Maximum Likelihood (D) methods. The related homeobox gene *Rx* (retinal homeobox) from *Drosophila melanogaster* serves as an outgroup. Gene names are prefixed with the initials of their respective species names. *Bf, Branchiostoma floridae, Pm, Petromyzon marinus, Mm, Mus Musculus, Sc, Scyliorhinus canicula, Dr, Danio rerio, Dm, Drosophila melanogaster, Sp, Strongylocentrotus purpuratus, Pl, Paracentrotus lividus, Ce, Caenorhabditis elegans, Mg, Myxine glutinosa.*
(TIF)

Figure S2 **Clustal alignments of** ***Runx*** **(A) and** ***Alx*** **(B) genes used to generate the trees in Figure S1.** Only the sequences spanning the highly conserved DNA binding domains are shown for each alignment.
(TIF)

Figure S3 **Expression of** ***RunxA, RunxB, Barx,*** **and** ***Alx*** **in larvae at 35 days post-fertilization.** Expression patterns are essentially the same as those seen earlier at st. 30. Side view (A) and ventral view (B) of *RunxA* mRNA distribution showing weak expression in pharyngeal endoderm. Side view (C) and ventral view (D) of *RunxB* mRNA distribution showing weak expression in pharyngeal endoderm. Side view (E) and ventral view (F) of *Barx* mRNA distribution showing persistent expression in the medial aspect of the posterior pharyngeal arches (arrowhead) and the ventral portion of the first pharyngeal arch (arrow). Side view (G) and ventral view (H) of *Alx* expression in the dorsal and ventral aspects of the branchial basket (asterisks), dorsal fin (arrowhead), and dorsal first arch (arrow).
(TIF)
