# Supplementary figures and images for: A New Mechanistic Scenario for the Origin and Evolution of Vertebrate Cartilage
Source: PLoS One. 2011 Jul 22;6(7):e22474. doi: 10.1371/journal.pone.0022474 (PMC3142159; doi:10.1371/journal.pone.0022474)

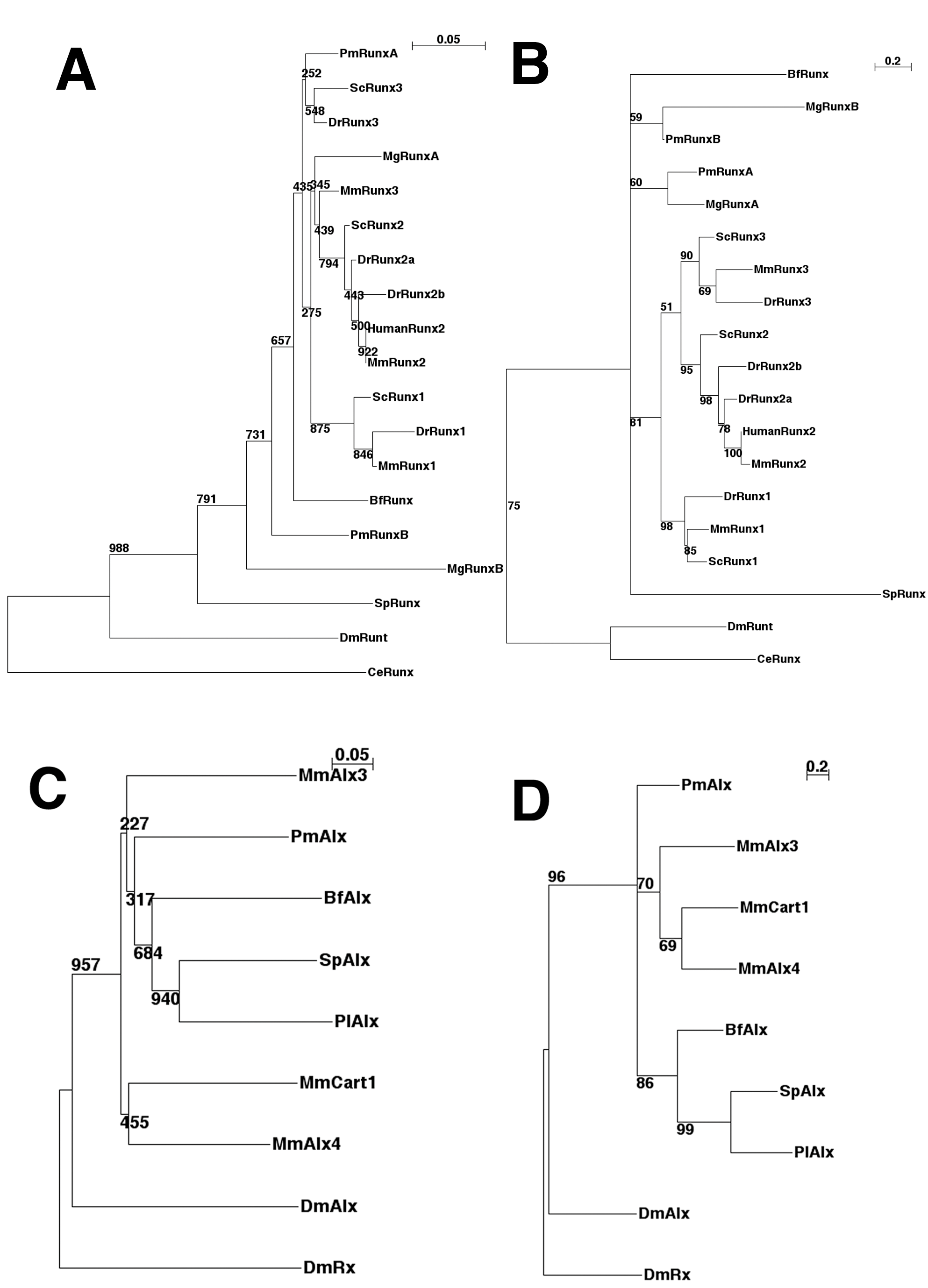

Supplement: Figure S1 — Phylogenetic analysis of lamprey Runx and Alx genes. Lamprey RunxA and RunxB group with deuterostome Runx/Runt homologs with high confidence values using either the Neighbor-Joining (A) or Maximum Likelihood (B) methods. Lamprey Alx groups with deuterostome Alx/Cart homologs with high confidence values using either the Neighbor-Joining (C) or Maximum Likelihood (D) methods. The related homeobox gene Rx (retinal homeobox) from Drosophila melanogaster serves as an outgroup. Gene names are prefixed with the initials of their respective species names. Bf, Branchiostoma floridae, Pm, Petromyzon marinus, Mm, Mus Musculus, Sc, Scyliorhinus canicula, Dr, Danio rerio, Dm, Drosophila melanogaster, Sp, Strongylocentrotus purpuratus, Pl, Paracentrotus lividus, Ce, Caenorhabditis elegans, Mg, Myxine glutinosa. (TIF) [file pone.0022474.s001.tif]

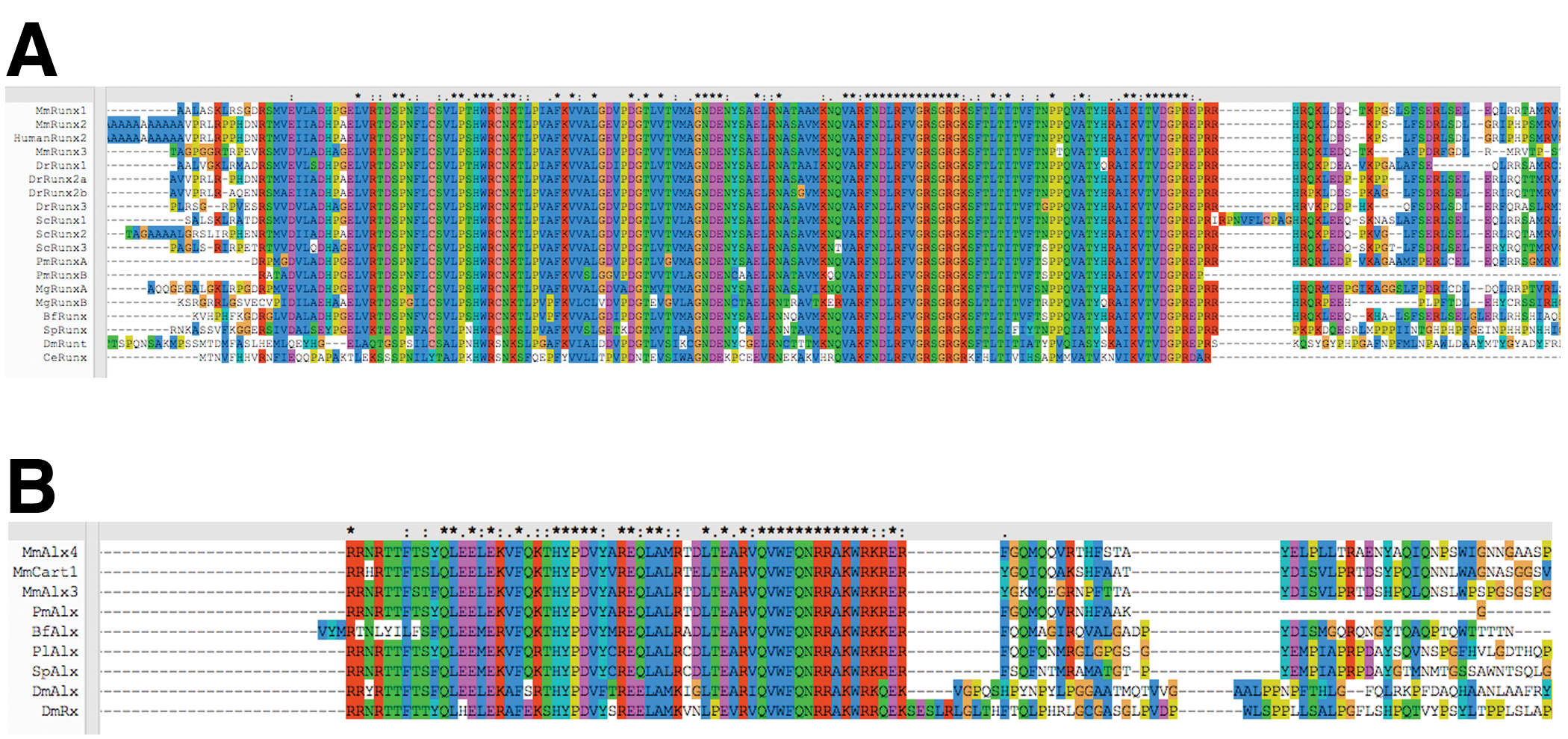

Supplement: Figure S2 — Clustal alignments of Runx (A) and Alx (B) genes used to generate the trees in Figure S1. Only the sequences spanning the highly conserved DNA binding domains are shown for each alignment. (TIF) [file pone.0022474.s002.tif]

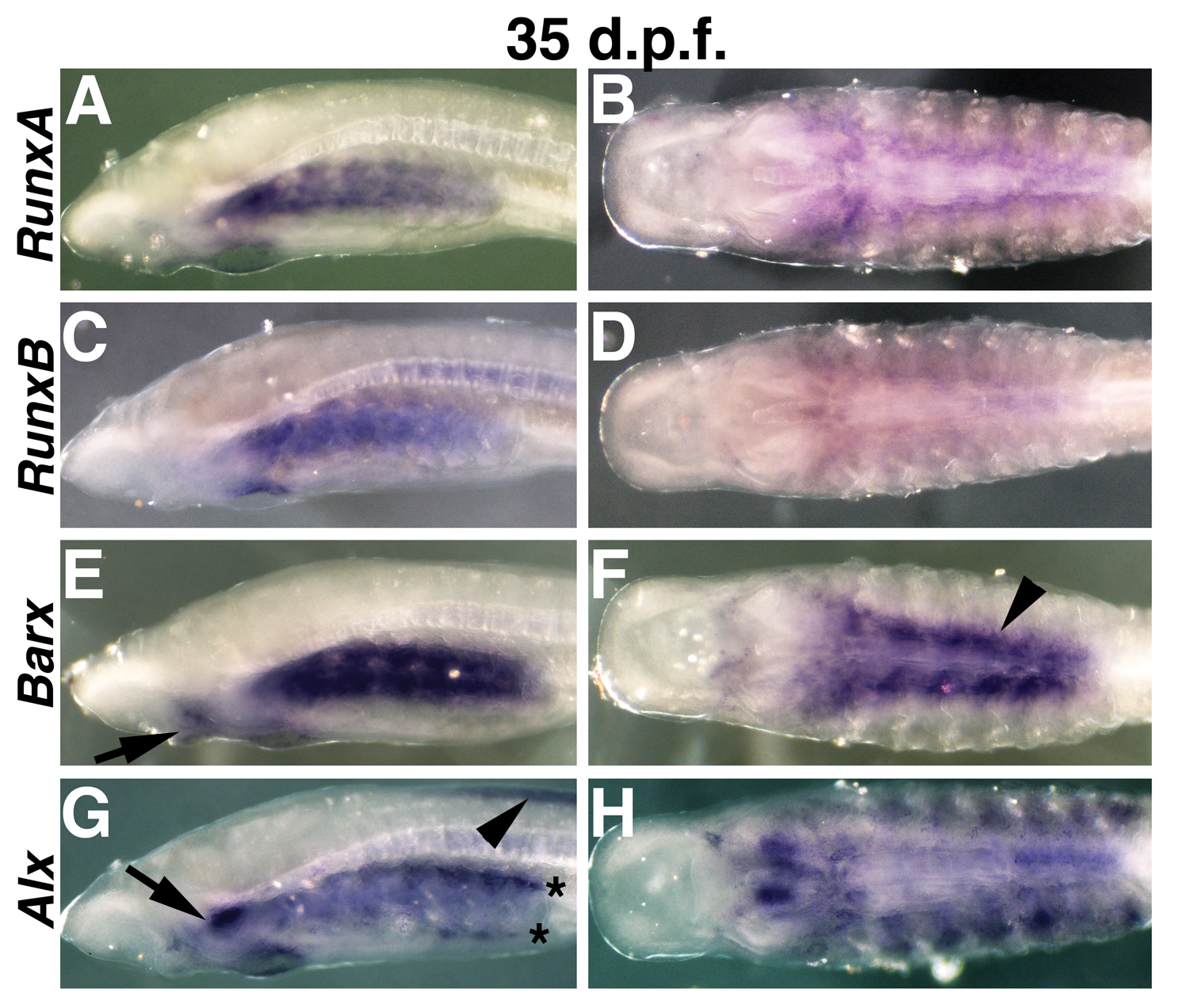

Supplement: Figure S3 — Expression of RunxA, RunxB, Barx, and Alx in larvae at 35 days post-fertilization. Expression patterns are essentially the same as those seen earlier at st. 30. Side view (A) and ventral view (B) of RunxA mRNA distribution showing weak expression in pharyngeal endoderm. Side view (C) and ventral view (D) of RunxB mRNA distribution showing weak expression in pharyngeal endoderm. Side view (E) and ventral view (F) of Barx mRNA distribution showing persistent expression in the medial aspect of the posterior pharyngeal arches (arrowhead) and the ventral portion of the first pharyngeal arch (arrow). Side view (G) and ventral view (H) of Alx expression in the dorsal and ventral aspects of the branchial basket (asterisks), dorsal fin (arrowhead), and dorsal first arch (arrow). (TIF) [file pone.0022474.s003.tif]
